# Increased release of glycine from endometrial mesenchymal stem cells in women using oral contraceptives might possibly limit the lytic death of surrounding cells in endometrium

**DOI:** 10.5935/1518-0557.20260042

**Published:** 2026

**Authors:** Caglar Berkel

**Affiliations:** Tokat Gaziosmanpasa University, Department of Molecular Biology and Genetics, TOKAT, Türkiye

Dear Editor,

In a very recent study published in *JBRA Assisted Reproduction*, Rochetti *et al*. (2026) reported that exposure to oral contraceptives (OCs) influences the secretome of endometrial mesenchymal stem cells (EnMSCs), which then potentially impacts endometrial plasticity. Endometrial mesenchymal stem cells in this study were collected from menstrual shedding of women of reproductive age, and then cultured for 3 passages (Rochetti *et al*., 2026). Culture media was collected at the end of each passage by the authors for further secretome analysis by quantitative metabolomics. Among other metabolites quantified in the culture media from two groups (OC and non-OC groups), the authors found that glycine concentration is higher in the OC group than in non-OC group (Rochetti *et al*., 2026). In other words, in culture, endometrial mesenchymal stem cells collected from women using oral contraceptives were found to secrete higher levels of glycine compared to those from women not using oral contraceptives (Rochetti *et al*., 2026).

Since glycine, as a cytoprotectant, is known to suppress plasma membrane rupture and lytic cell death, thus limiting inflammation (Borges *et al*., 2022), I here hypothesized that increased release of glycine from endometrial mesenchymal stem cells in women using oral contraceptives might increase the resistance of other endometrial mesenchymal stem cells (and possibly other cell types) in the same microenvironment to lytic cell death, by preserving cellular integrity. Mechanistically, glycine prevents the oligomerization of NINJ1 (ninjurin-1), a protein which mediates plasma membrane rupture and the loss of plasma membrane integrity following certain cell death mechanisms such as pyroptosis (Borges *et al*., 2022; Kayagaki *et al*., 2021). This potential glycine-mediated resistance to lytic cell death in EnMSCs and surrounding cells in women using OCs might then result in decreased endometrial inflammation. In other words, increased secretion of glycine from EnMSCs due to OCs might possibly limit NINJ1-mediated plasma membrane rupture and the release of large intracellular pro-inflammatory molecules to the extracellular space in nearby cells, ultimately limiting the endometrial inflammation. However, these hypotheses should be experimentally tested to determine whether glycine levels released from EnMSCs in women using oral contraceptives are indeed sufficient to limit lytic death of surrounding cells in the endometrium.
